# The use of personal digital assistants for data entry at the point of collection in a large household survey in southern Tanzania

**DOI:** 10.1186/1742-7622-4-5

**Published:** 2007-06-01

**Authors:** Kizito Shirima, Oscar Mukasa, Joanna Armstrong Schellenberg, Fatuma Manzi, Davis John, Adiel Mushi, Mwifadhi Mrisho, Marcel Tanner, Hassan Mshinda, David Schellenberg

**Affiliations:** 1 Ifakara Health Research & Development Centre, PO Box 78373, Dar es Salaam, Tanzania; 2 Department of Infectious and Tropical Diseases, London School of Hygiene and Tropical Medicine, London, UK; 3 National Institute of Medical Research, PO Box 9753, Dar es Salaam, Tanzania; 4 Swiss Tropical Institute, Socinstrasse, 57, Postfach CH 4002, Basle, Switzerland

## Abstract

**Background:**

Survey data are traditionally collected using pen-and-paper, with double data entry, comparison of entries and reconciliation of discrepancies before data cleaning can commence. We used Personal Digital Assistants (PDAs) for data entry at the point of collection, to save time and enhance the quality of data in a survey of over 21,000 scattered rural households in southern Tanzania.

**Methods:**

Pendragon Forms 4.0 software was used to develop a modular questionnaire designed to record information on household residents, birth histories, child health and health-seeking behaviour. The questionnaire was loaded onto Palm m130 PDAs with 8 Mb RAM. One hundred and twenty interviewers, the vast majority with no more than four years of secondary education and very few with any prior computer experience, were trained to interview using the PDAs. The 13 survey teams, each with a supervisor, laptop and a four-wheel drive vehicle, were supported by two back-up vehicles during the two months of field activities. PDAs and laptop computers were charged using solar and in-car chargers.

Logical checks were performed and skip patterns taken care of at the time of data entry. Data records could not be edited after leaving each household, to ensure the integrity of the data from each interview. Data were downloaded to the laptop computers and daily summary reports produced to evaluate the completeness of data collection. Data were backed up at three levels: (i) at the end of every module, data were backed up onto storage cards in the PDA; (ii) at the end of every day, data were downloaded to laptop computers; and (iii) a compact disc (CD) was made of each team's data each day.

A small group of interviewees from the community, as well as supervisors and interviewers, were asked about their attitudes to the use of PDAs.

**Results:**

Following two weeks of training and piloting, data were collected from 21,600 households (83,346 individuals) over a seven-week period in July-August 2004. No PDA-related problems or data loss were encountered.

Fieldwork ended on 26 August 2004, the full dataset was available on a CD within 24 hours and the results of initial analyses were presented to district authorities on 28 August. Data completeness was over 99%.

The PDAs were well accepted by both interviewees and interviewers.

**Conclusion:**

The use of PDAs eliminated the usual time-consuming and error-prone process of data entry and validation. PDAs are a promising tool for field research in Africa.

## Background

Field surveys are an integral part of health research, but standard approaches to data recording and processing are cumbersome, time-consuming and error-prone. The proper documentation of subjects' responses depends on specially trained interviewers understanding the logical flow of questions and any skip patterns. Internal inconsistencies may arise from interviewer error, and erroneous numerical values, for example those outside of possible ranges, may be written on a form. Some errors or omissions may be detected at the end of each working day when questionnaires are supposed to be painstakingly checked, one by one, for completeness and accuracy by a field supervisor. Though correction of responses may no longer be possible, a supervisor can draw the errors to the attention of the interviewer in the hope that the chance of repeat errors will be reduced. Upon survey completion, the paper forms are sent for double data entry by independent data entry personnel, before resolution of any discrepancies between the two entries by a third person. This process is intended to ensure that the data available in the database are a true reflection of the information on the forms, but the original forms are usually archived in case of subsequent query. Data cleaning can then start, though by this stage it is not usually possible to correct any data omissions, logical, range or other internal inconsistencies and 'missing value' codes are substituted. There may thus be a considerable time-lag between data collection and the availability of incomplete data for analysis. This is nevertheless the standard approach to data processing for most large-scale health surveys[1,2].

The development of Personal Digital Assistants (PDAs) and related software has made data entry at the point of collection (DEAPOC) a realistic prospect. DEAPOC enables instantaneous range, consistency and logical checks at the time of data entry, enhancing data quality and dramatically reducing the time until data are available for cleaning and analysis. We report the development and use of DEAPOC in the context of a large household survey in remote areas of southern Tanzania.

## Methods

### Survey and study area

The aim of the survey was to generate baseline information on health and survival in young children prior to a cluster-randomized evaluation of a new approach to malaria and anaemia control. Specifically, infant mortality estimates were required from each of the 24 administrative divisions in the project area to allow pre-randomisation stratification. The survey was conducted in July-August 2004 and targeted 21,600 households. The five rural districts in the study area (Lindi Rural, Ruangwa and Nachingwea of Lindi region, and Newala and Tandahimba of Mtwara region) cover an area of approximately 20,000 km^2 ^near southern Tanzania's border with Mozambique. The area is relatively undeveloped with high mortality, malnutrition and illiteracy rates. Most people live in simple mud-walled, thatched houses and depend on subsistence farming, fishing or small scale trading. Mains electricity is available in less than 1% of households and most villages have no electricity. There are only two tarmac roads in the area and travel via the many dirt roads is problematic during the rainy season.

### The data processing system

#### 1. Hardware

Interviewers used Palm m130 PDA units (Palm Inc, 950 W. Maude Ave, Sunnyvale, CA 94085), each costing approximately $100, to enter data directly into databases via the standard 160 × 160 passive matrix STN coloured touch sensitive screen. PDAs measured 4.8 × 3.1 × 0.9 inches, weighed 5.4 oz and comfortably rested in the palm of a hand. The rechargeable internal lithium ion battery lasted for at least two full working days between recharges. The units' 33 Mhz Motorola Dragonball VZ processor ran the Palm OS(r)4.1 operating system. In addition to the standard 8 MB memory, a 16 MB SD memory expansion card was installed. Data were transferred from the PDA to a laptop computer via a USB link cable and synchronising software (Palm TM Desktop for windows 4.0.1), supplied with the PDAs.

#### 2. Software

The data capture software was Pendragon Forms 4.0 [Pendragon Software Corporation, 1580 S. Milwaukee Ave Suite 515, Libertyville, IL 60048, USA] which consists of three components: (i) The Desktop Designer and Database, a Microsoft Access application that includes a form designer and tools for viewing, managing and exporting data; (ii) the Sychronization Conduit, which transfers form design, data and look-up lists from the computer to the PDA and data from the PDA to the computer during synchronization; (iii) the Handheld Application, a program run on the PDA during the data entry process. Pendragon Forms 4.0 allows scripts containing logical statements to be attached to questions and evaluated at the time of data entry. These scripts can be used to display customized error messages, for example "This question must be answered before you can continue", "Date of vaccination precedes date of birth", or "The cluster number cannot exceed 720". Scripts can also be used to hide a series of questions if a skip pattern should be executed. Pendragon supports more than one user, thus one form can be sent to different handheld devices and all the records from different users can be stored in the same database. Each record contains a unique identifier, so individual records can be tracked and those from a single household can be readily linked.

#### 3. Form design

**Figure 1:**
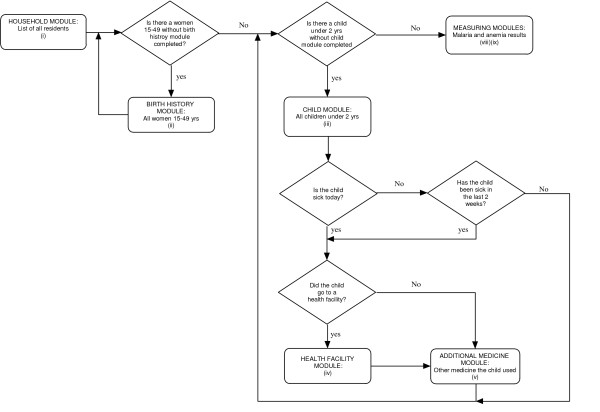
Logical flow of the questionnaire.

Nine modules of questions were used in the survey (figure 1): (i) the Household Module recorded information such as demographic details of household members, information on assets and latitude and longitude; (ii) the Birth History Module recorded information on all live births, and was completed for all women aged 15 to 49 years; (iii) the Children's Module recorded health-related information, and was completed for children under two years of age; (iv) the Health Facility Module documented information about any health facility to which children who had been sick within the preceding two weeks were taken; (v) an Additional Medicine Module recorded information on drug treatments other than those dispensed from a health facility for children sick within the preceding two weeks; (vi) the Under Two Invitation and (vii) the Over Two Invitation modules displayed summary information about each individual in the household, facilitating the completion of a written invitation to attend a measuring station. A unique sample number was entered into the PDA and onto the invitation to enable subsequent linkage with blood test results captured on a different PDA at the weighing station, situated in the middle of the village. Finally, modules (viii), the Under Two Measurement Module, and (ix), the Above Two Measurement Module, were used at the central weighing station to record results.

A household module was to be completed for each household, and Birth History modules for each woman aged 15 to 49 years. In a subset of 5000 households, children under two years should have had the Children's and Health Facility modules completed, including a large number of questions about the nature of any illness experienced in the preceding two weeks. Additional Medicine Modules should also have been completed for children who had been sick. In households selected for the children's modules, every resident was given an invitation to attend the measuring station.

The Household Module was manually loaded by an interviewer on arrival at each house. All questions were presented in Swahili, the most widely-spoken language in Tanzania. The name, date of birth and sex of each resident were entered before the PDA automatically loaded the Birth history, Children's and Invitation Modules the required number of times. The Children's Module controlled the loading of the Health Facility and Additional Medicine modules. Measurement Modules were loaded manually by the interviewers based at the measuring station.

Control mechanisms ensured that the different forms were loaded the required number of times and for the appropriate people, and that linkage information was maintained across the different forms. For example, if there were two women aged 15 to 49 years, the program would copy automatically-generated identification numbers for each woman, together with their names, to another table and would set a counter for the number of birth history module forms to be loaded. On completion of the Household Module, the Birth History Module would automatically be loaded, governed by the counter which was reduced by one each time the form was loaded. On the name field within the birth history module, the interviewer would choose from a lookup list containing the names of all women aged 15 to 49 years within that particular household. By doing so, the woman's details would be copied automatically to the appropriate field of the Birth History Module. Additional Birth History Modules would be loaded until the counter diminished to zero.

Most fields had checks built in for control purposes. Some additional check questions were included: for example, when the interviewer had entered a date of birth and gender, a script would compute the age of the person and, depending on the age, the next question would either be "Is this a woman aged between 15 and 49 years" (all people aged 15 years and above) or "Is this a child under two years" (for all people below 15 years). In the event of an unacceptable response (for example a response inconsistent with an earlier value, or an out of range value), an error message would be displayed (for example "Date of death is before date of birth") and the interviewer could not proceed until the error had been corrected.

The link between demographic and health information, collected at the house, and the results of tests from the measuring station, depended on a unique, individually generated sample number. To reduce the chance of mistakes on entry of the sample number, a script ran a check sum function to validate the number and to alert the interviewer to any mistake in data entry.

Programming of the forms was completed by a data manager with less than three years diploma-level training. The process from start of familiarization with Pendragon, using the on-line user guide, to pilot testing took about six weeks.

#### 4. System piloting

A five-day pilot with eight interviewers and two field co-ordinators was conducted in May 2004. Most interviewers had no prior experience of computers and hence the pilot assisted in the development of the approach to training. Interviews were conducted in class and in the field and valuable lessons were learnt, including how to improve the phrasing of questions and linkage of the different modules. The interviewers participating in the pilot were then able to assist in the training of much larger numbers of interviewers for the main survey.

#### 5. Data security

The form was designed and linked such that, on completion of a set of modules at a household, the records would be copied to the SD card and the record locked on the PDA. Forms could not be loaded to allow data entry if the SD-card was not present, was improperly inserted or set to read-only mode. Interviewers were not able to see or amend a record once it had been copied to the SD card, although the record was retained on the PDA. Every supervisor had a laptop, which they used for daily synchronization of data from the PDAs before burning a back-up to CD. The CDs were collected every few days by a support vehicle and deposited in a secure store. The data on the laptop were exported to secondary files that were used to run automated checking routines (see below). Supervisors wrote in ordinary notebooks to record solutions to any problems identified, but were unable to edit or clean the data themselves.

### Survey logistics

#### 1. Training the survey team

Prospective interviewers were identified from the survey area and invited to a two-week training course. Most had four years' secondary school education and very few had prior experience with computers. The first PDA training session was on the afternoon of day one, when trainees were told about care of PDAs and introduced to some key features (e.g. on/off button, home icon, battery indicator, use of on-screen keyboard). Trainees were then introduced to several PDA-based 'forms' to familiarize them with writing, deleting and moving back and forth between questions. They were encouraged to type *"The quick brown fox jumped over the lazy dogs*", a sentence containing all the letters of the alphabet, by the lure of a prize for the trainee who correctly typed it fastest, according to an automatic timer in the form. Trainees were then introduced to a form that documented information about themselves (the "CV form"). This introduced them to entering dates, digits and correcting mistakes, as well as the use of pop-up lists (e.g. to select gender) and look-up lists, where the first selection decides which options are available on subsequent lists. On this first day of training, no validations or restraints were imposed, to avoid demoralization. However, on the second day of training, logical and range checks were introduced to the CV form so that trainees could start to see the power of the PDA. The first survey form was also introduced, after an opportunity to familiarize themselves with a paper version. A projected image of the trainer's PDA screen (via Presenter-to-go by Margi System 39465 Paseo Podre, Parkway, Fremont, CA 94538) enabled trainees to see where they were supposed to be at any particular moment, what should happen and what error messages they might get. After reviewing the form in plenary, small groups were formed and trainees took it in turns to ask the questions of a single respondent (one of those who had participated in the PDA pilot) who acted as the head of a household. Pre-determined sets of responses were used and data routines developed to summarize discrepant responses from trainees and thus facilitate objective assessment of trainees' performances.

As training and piloting continued during the second week interviewers were taught how to recharge their PDAs from mains electricity and a solar charger (Select Solar Ltd, 2 Eysey, Cricklade, Swindon, UK). During the field work, each team also had a PDA car charger. Throughout the training, the importance of interview technique was repeatedly stressed, frequent accompanied interviews performed and guidance given to interviewers as necessary. Interviewers were also taught to use a variety of other devices including battery-powered Global Positioning System (GPS) units (Garmin GPS 12, GARMIN International, Olathe, USA) and equipment to check for anaemia and rapid diagnostic tests for malaria.

#### 2. Survey logistics

At the end of training, 104 of the 120 trainees were split into 13 teams of seven interviewers, each with a supervisor and a vehicle. Teams were assigned villages in which to conduct the survey and the supervisor visited each village the day before the survey to identify 30 households at random and to leave an information sheet at each selected house. The supervisor assigned a number to each household and allocated households to individual interviewers. Interviewers sought written informed consent from the heads of households. The consent letter specifically included a few words mentioning the use of PDAs. Where consent was given, the interviewers proceeded with questioning and manually entered the household's geographic co-ordinates as indicated on the GPS unit. Before leaving each household the interviewers completed a hand-written form documenting whether the household had been willing to participate in the survey, the number of women of child-bearing age, and the number of children under two years of age and whether or not they had been sick. At the end of each day, a manual summary of this information for the whole cluster was prepared by the supervisor on a Cluster Summary Sheet and compared with an electronic summary derived from the data actually entered into the PDAs.

Two support vehicles, each with a field co-ordinator and data support technician, as well as spare PDA units and other supplies, visited each team every few days. In addition, team supervisors had a mobile phone and could travel to an area with phone coverage to summon more urgent assistance if required. Interviewers were responsible for ensuring their PDA was fully charged at the beginning of each day. At the end of each day, PDAs were handed to the supervisor for data synchronization and back-up to CD. During routine support visits, the back-up CDs were collected from supervisors and the data technician would check if the supervisor had run a series of check routines every working day, review the synchronization logs to identify any problem with data transfer, and check if the supervisor had documented all the errors captured by the check routines and, where possible, their solutions.

### Quality control

Supervisors performed several accompanied interviews each day to ensure consistency of approach between interviewers. In addition, key questions from the survey were included in a separate, repeat interview completed by supervisors on randomly-selected households that had been interviewed the same day. The repeated questions were selected such that either the responses were unlikely to change between the initial and the supervisor's visit (for example data on members of the household, type of roof, ownership of mosquito net) or were of critical importance in determining large skip patterns (for example "Has the child been sick in the last two weeks?"). At the end of each day a program written in Microsoft Access was used to compare the two sets of responses, producing details of any discrepancies that could then be resolved.

Additional routines produced daily reports and allowed limited interrogation of the dataset to investigate problems. As all modules were ultimately controlled by the Household Module, the routine checked that a single household-level record was available for each of the 30 households in that day's cluster and provided details of missing or repeated households. The automated routine also produced a summary directly comparable with the supervisor's cluster summary sheet and could produce the same information at household level to facilitate identification of the interviewer and household where any discordance arose. The report also identified GPS readings that were out of range, enabling re-recording before the survey team moved on, and the number of women aged 15 to 49 years who had not been interviewed, usually because of absence from the home at the time of the interview, who could then be followed up. The routine provided information on interviewers' performance, which guided discussions during daily review meetings.

There was no need for the supervisor to review responses to individual questions, completeness of responses, observance of skip patterns, range checks or information linking the various modules to each other for a given house, as these were all taken care of automatically.

### Assessment of acceptability

Thirty interviews were conducted within a week of the end of the survey to assess perceptions and acceptability of the PDAs in the local community. In-depth, semi-structured interviews with key informants explored perceptions of the original interview and comparison with the interviewees' experience of other surveys. Respondents were asked if they had noticed anything unusual during the interview and, if not, whether they could recall how the interviewer had recorded their responses.

## Results

None of the trainees had major difficulties using the PDA, although some older trainees with poor eyesight reported difficulty reading the screen. However, no trainees failed to be appointed as interviewers because of difficulties using the PDA. Previous survey experience was not related to ease of use of PDA.

**Figure 2:**
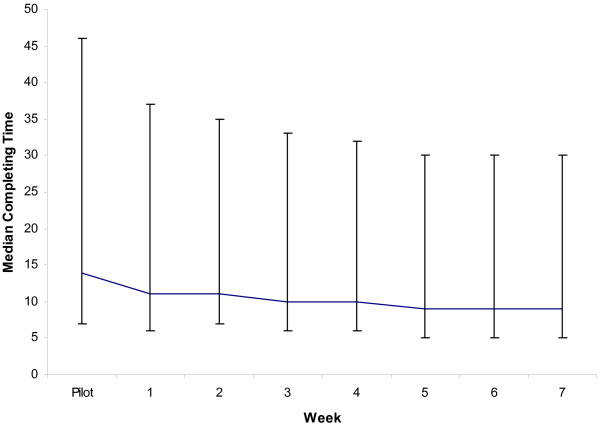
Median (5^th ^and 95^th ^centile) time to complete the household module during the training and survey.

Figure 2 shows the median time (with 5% and 95% centiles) taken to complete the household module during the training and survey. The household module took a median of 14 minutes (seven to 32 minutes) to complete during training, decreasing to a median of nine minutes (four to 21 minutes) during the survey. Initially there was a tendency for interviewers to focus on the PDA rather than the respondent, although interview technique was considered satisfactory in all staff before the end of training.

No hardware or software failures occurred on either the PDAs or laptops, and no data were lost during the course of the survey.

Data were collected from a total of 21,474 households. In 497 households (2%), nobody was present to give consent and in a further 91 (0.4%), the household head was not willing to participate. Data completeness was high. For example, a socio-economic score derived from a composite of eight household factors (ownership of radio, bicycle, animals, poultry; roof type; owner-occupier status; availability of electricity; normal cooking fuel) was available for 99% (n = 20,636) of households.

Fieldwork was completed on 26^th ^August 2004 and preliminary results presented to local leaders two days later.

### Acceptability

Although some interviewees did not volunteer anything unusual about the interview, most respondents said that they had noted the PDA after its mention in the consent letter. Several interviewees appreciated the interviewer having introduced them to the gadget during the consent procedure. Respondents reported discussions about the PDAs in the community after the interviewers had left. Most interviewees who had an opinion were positive. For example, a 30-year old man with primary-level education said "I was very happy to see a computer as it was my first time to see it. It simplified recording of our responses". An elderly man expressed his appreciation of having learnt what day of the week he had been born.

Interviewers and supervisors unanimously expressed their satisfaction using DEAPOC during the survey.

## Discussion

We have described the use of PDAs in a large household survey in remote areas of rural southern Tanzania. Training of interviewers with no prior computer experience proved straight forward and no major hardware problems were experienced during the survey. Completeness of data was very high and cleaning of the full dataset took less than one person-month to complete. There was a very high level of acceptance by the community and unanimous approval by interviewers, supervisors and field co-ordinators alike.

A number of factors probably help explain the high acceptability of PDAs in our survey. A key reason is likely to be the provision of information to interviewees. The consent letters were made available a day before the survey and the PDAs mentioned at this stage. Interviewers were encouraged to show the PDA during the formal consent procedure. We also attached importance to interviewers' behaviour and the need to show respect to the local community. One old man said of the interviewers "hawakuja kwa utemi" meaning that they did not come boasting and had explained themselves precisely.

A weakness of our report is that there is no direct comparison with a paper-based version. Reassuring comparisons have been conducted in other settings [3-5], although further head-to-head comparisons with alternative approaches for data capture would be useful. However, there appear to be marked benefits of PDA use in the field. Our data had a high level of internal consistency and completeness and were available much more quickly than would have been the case with traditional paper-based approaches.

A major feature of the PDA-based approach is the increased need for careful pre-training preparation than might be the case for a paper-based survey. In particular, time is needed to pre-program acceptable ranges for numeric variables, to program skip patterns, to check for logical consistency in responses and to thoroughly test that the routines work. Once done, however, there are marked reductions in the extent of checking necessary by supervisors during the survey, freeing up time for other quality control activities, and reductions in the amount of data cleaning required once field activities are complete. Although the large part of the questionnaire and programming are finalized pre-training, minor modifications to the questions can still be made during the course of the training as time-consuming and costly print runs are not required to effect any changes. Furthermore, DEAPOC does away with the need to transport, account for and archive large numbers of bulky forms. The time required to train interviewers was not increased as a result of DEAPOC and the observation that the time required to complete interviews did not reduce dramatically after the pilot period (figure 2) suggests that there would be little to gain by extending the duration of training.

The need to re-charge the internal battery is an obvious concern in remote rural settings with no mains electricity supply. However the solar chargers performed well in our survey, apart from the contacts of a few connectors (solar charger to PDA) being broken and becoming unusable during the two months in the field. Otherwise, we had no failures of electronic devices despite the hot and dusty conditions and unfamiliarity of most survey staff with such delicate instruments. The specific sessions included in the training on appropriate care and carrying of the PDAs in locally-made carrying cases may have helped. An initial irritation resulted from insufficiently careful calibration of some of the PDA screens. As a result, some of the new trainees were perplexed not to have their inputs accepted by the units.

An unusual logic was required to maximize the quality of data collected, focusing on information from the household level. Once residents' information had been entered all subsequent loops and responses were taken care of automatically. However, it was of critical importance to ensure that the house and cluster numbers, dates of birth and sex of household members had been entered correctly. To this end, a number of internal check questions and a series of data checking routines proved useful. The output of all routines was logged, providing additional opportunities for the survey co-ordinators to ensure that supervisors were using the data routines. The ability to synchronize the PDAs and run data checking routines in the villages also made it possible to identify and correct errors (e.g. incorrectly entered GPS co-ordinates) that would otherwise have resulted in missing values.

There are a number of potential disadvantages to DEAPOC. The Pendragon-based system we used does not generate a formal audit trail, which is a requirement for compliance with Good Clinical Practice guidelines. GCP-compliant software is now available for data management on PDAs, but the databases generated tend to become rather large and cumbersome to work with. As a result, further software development is likely to be necessary before PDA-based data collection will be acceptable to regulatory agencies.

Data security is sometimes raised as a concern in DEAPOC. Although we lost no data during our survey we were aware that if, for example, a PDA was dropped in water after a day's work but before synchronization, that day's data may be lost. Concerns have also been expressed that a stolen PDA might risk confidential data becoming available to others. We reduced the chance of this by locking records on completion of the Household Module (before leaving the house) and making it impossible to view records on the PDA once they had been locked.

The initial capital investment is clearly proportional to the size of the survey and sensitive to the number of interviewers per survey team. The single most expensive item was the laptop, held by each supervisor. We used low-specification equipment; the most inexpensive PDAs capable of running Pendragon forms, bottom-of-the-range laptops able to synchronize with PDAs and burn CDs for data backup, and independent GPS units (rather than the more expensive GPS units integrated into PDAs). We reckon the equipment we bought would last up to five years if used for 10 weeks per year and, on this basis, cost approximately $60 per supervisor per week and $11 per interviewer per week. Including the costs of programmers' time, training and piloting, the data processing costs in our survey worked out at $0.85 per household, comparing favourably with the $1.25 per household for a similar, paper-based survey conducted in 2003.

Since the survey reported here we have also used DEAPOC for time and motion studies in health facilities, for longitudinal follow-up studies evaluating the efficacy of antimalarial drugs and for further household surveys. New programming innovations (such as those present within the Pendragon service pack four) have further enhanced the power of DEAPOC. In our experience, DEAPOC represents a cost-effective and time-saving approach to enhance the quality and completeness of data.

## Competing interests

The author(s) declare that they have no competing interests.
